# Incidence and Etiology of Stroke among Hospitalized Children: A Case-Series Study

**Published:** 2019

**Authors:** Zarin Taj KEIHANI DOUST, Fatemeh NOORI SARI, Parvin AKBARI ASBAGH, Zahra FARAHANI, Fatemeh TEHRANI, Mamak SHARIAT

**Affiliations:** 1 **1.Maternal & Child Health Specialist, Maternal-Fetal Neonatal Research Center, Tehran University of Medical Sciences, Tehran, Iran**

**Keywords:** Stroke, Infant, Adult children, Cerebral blood flow

## Abstract

**Objectives:**

Stroke is a sudden blockage or rupture of brain vessels resulting neural defect or impairment. We aimed to investigate the incidence and causes of stroke in hospitalized children (Tehran-Iran, 2008-2013).

**Materials & Methods:**

This case series study was carried out in pediatric ward of tertiary care Vali-Asr Hospital, Imam Khomeini Hospitals Complex (Tehran- Iran) from 2008 to 2013. One month to 15 yr old admitted children due to stroke were enrolled into this case series study. Diagnosis was confirmed with brain imaging. Participants' demographic data, potential risk factors and neuroimaging findings were obtained from Hospital Reporting System. Recorded data were studied and considered regarding the incidence of stroke and its causes. Indeed we investigated cardiological causes as well as different items related to hematological disorders.

**Results:**

Of 20000 admitted subjects in Imam Hospital during 5 yr, stroke was diagnosed in 15 cases. The incidence among the population study was 0.75 per 100000 children. Stroke was more frequent in males than females ($\frac{1.14}{1}$). The most common age of stroke was 4-6 yr and the mean age of stroke was 58.8 months equal to 4.9 year. The most frequent stroke was hemorrhagic stroke (26%), followed by vascular (20%) and coagulopathy disorders (20%).

**Conclusion:**

The incidence of stroke in children was 0.75 per 100000. Hemorrhagic stroke due to major trauma, coagulopathy and vasculopathy were observed as most frequent causes that necessitate implementing some strategies for prevention, earlier diagnosis, and treatment.

## Introduction

Stroke is rapidly declined of cerebral blood flow resulting impairment of brain function (1). The incidence of childhood stroke is about 13-16 in 100000 (2). In the United States, stroke with mortality rate of 0.34 deaths per 100000 is one of top 10 causes responsible for death among children (3). 

The causes of stroke in children are different from adults. Thrombophilia, sickle cell anemia, infections, acquired or congenital emboligenic heart diseases were reported as important causes. Stroke in children may lead to death, cognitive-motor impairment and long-lasting seizure (24 h or longer) (2).

Congenital heart disease is reported as a profound risk factor for ischemic and hemorrhagic stroke (4). The rate of ischemic stroke in children with CHD is about 10-fold higher than age and sex-matched children in the control group (5). Moreover, the frequency of vaso-occlusive stroke may increase during cardiac surgery based on child's age, duration of bypass or reoperation (6). Coagulopathy and thrombophilia in combination with other factors are reported a potential risk factor for arterial ischemic stroke. Genetic factors like Moyamoya Syndrome and Sickle cell disease are also responsible for 6% and approximately 1% of overt stroke, respectively (7). Metabolic diseases and trauma resulting infarction are uncommon; however, these conditions are important for the pediatric population (1).

Stroke is known as an important cause of mortality (18%) or lifelong disability in two-thirds of the children (1-3). We aimed to evaluate the incidence and main underlying causes of stroke which would be of value in implementing strategies to decrease morbidity and mortality rate as well as economic burden on families and society. 

## Materials & Methods

This case series study was carried out in Pediatric Ward of tertiary care Vali-Asr Hospital, Imam Khomeini Hospitals Complex (Tehran- Iran) from 2008 to 2013. All admitted patients due to stroke; aged one month to 15 yr old entered the study. Inclusion criteria were presence focal acute neural impairment confirmed by brain imaging. Neonatal strokes were excluded due to variable etiologic patterns. Participants' demographic data and potential risk factors including gender, age, clinical presentation and neuroimaging findings were obtained from Hospital Reporting System. Underlying disease and causes (prenatal problems, congenital heart disease, hematologic disorders, infection of CNS and trauma) were also collected and studied. Recorded data were considered regarding frequency of stroke and the causes. Frequency was presented as mean ± SD. 

This manuscript was taken from a thesis with ID 22664. Our study was approved by the institutional Review Board of Tehran University of Medical Sciences according to Helsinki declaration. The gathered data were confidential. 

## Results

Of 20000 admitted children in Vali-Asr Hospital during 5 yr led study, stroke was diagnosed in 15 cases. The incidence rate among the population study was 0.75 per 100000 children. Stroke was more frequent in males than females; male/female ratio was 1.14/1. The incidence of stroke in males and females were 0.40 and 0.35 per 100000, respectively. The most common age of stroke was 4-6 yr and the mean age of stroke was 58.8 months equal to 4.9 yr. The most frequent cause of stroke was hemorrhagic stroke due to trauma, followed by vascular and coagulopathy disorders. Of 4 cases with hemorrhagic stroke, stroke in 3 of them was due to child sexual abuse and a case due to severe car accident. Demographic data and factors associated with stroke in 15 patients are shown in Table 1.

## Discussion

Although pediatric stroke is not common, it significantly effects on morbidity- mortality rate and quality of life (8, 9). This retrospective population-based study was conducted to determine the incidence and risk factors for pediatric stroke. Based on our results, the incidence of stroke was 0.75 per 100000 among the population study. Former study from Iran has shown a higher rate; the annual incidence of children stroke (<15 yr) in 2006 was reported 1.83/100000 (10). The reported incidence of pediatric stroke (<15 yr of age) in Hong Kong between 1998 and 2001 was higher than in our population (2.1 cases per 100000) (3). The incidence of pediatric stroke (ischemic and hemorrhagic stroke) was reported 2.70/100000 child-yr in Austria, from 1984-2005 (11). The incidence of stroke varies in different countries based on some factors like the ethnic background, age-dependent references and the number of population study (8).

**Table 1:** Demographic data and factors associated with stroke in 15 patients

**Variables**	**n (%)**
**Sex**	
Male female	8 (53) 7 (47)
**Age(yr)**	
1-3 4-6 7-9 10-12 13-15	6 (40) 7 (46) 0 1 (6.66) 1 (6.66)
**Causes**	
- Congenital Cardiovascular -Vascular disease; (Artery-Vein malformation and hemangioma) -Hemorrhagic stroke -Coagulopathy; (Protein C,S deficiencies and thromboembolism) -Post surgery -Congenital metabolic disease; (Mitochondrial disease and lactic acidosis) -PROM* -Idiopathic	1 (6.66) 3 (20) 4 (26.6) 3 (20) 1 (6.66) 1 (6.66) 1 (6.66) 1 (6.66)

* premature rupture of the membranes

**Table 2:** Comparison of male/female ratio in different studies

Variables	Present study	Report by Tavassoli et al. (14)	Report by Deng et al. (12)	Report by Shi et al. (13)	Report by Chung et al. (3)
Male	53%	60%	60%	57%	56%
Female	47%	40%	40%	43%	44%
Male/Female ratio	1.14/1	1.5/1	1.5/1	1.32/1	1.27/1

**Table 3:** Comparison of causes in different studies

Causes	Present study	Report by Tavassoli et al. (14)	Report by Shi et al. (13)	Report by Chung et al. (3)	Report by Simma et al. (11)
Trauma	26.6%	5%	11%	-	0
Vascular disease	20%	2.5%	0	26%	85%
Coagulopathy	20%	7.5%	0	28%	50%
Heart disease	6.66%	17.5%	0	30%	0
Hb disorders	0	0	0	-	0
Metabolic disorders	6.66%	0	0	-	0
Post surgery	6.66%	0	0	-	0
Idiopathic	6.66%	40%	32.5%	12%	13.6%

Our results also showed that stroke was more frequent among males than females. Inconsistent with our results, more than 60% of stroke was observed in male gender (12). In a study, of 157 children with arterial ischemic stroke; male/female ratio was 1.4:1 (13). This ratio was compared among different studies and is shown in Table 2.

The most common age of stroke in the present study was 4 to 6 year, consistent with other a mean age of 5.5 yr among 17 cases with stroke (10). The most common age of stroke was 2 to 5 yr old among 40 Iranian children aged 3 months to 14 yr old (14). Of the 27 children aged between 6months and 15 yr with stroke, the average age was 6.91 (±2.08) year (2). On the other hand, stroke was more common in children less than 1 yr (51.35%) (8).

Traumatic hemorrhagic stroke was the most frequent cause of stroke in our pediatric population (26.6%). This rate is inconsistent with another study where head trauma and arterial dissection reported in 25.7% of pediatric brain infarction (15). On the other hand, spontaneous intraparenchymal hemorrhage, non-traumatic subarachnoid hemorrhages, and arterial ischemic stroke are the most frequent causes of stroke with incidence 1.5–2.9 per 100.000 children (16). 

Of 94 pediatric strokes, 14 strokes (28%) were hemorrhagic (3). Moreover, hemorrhagic strokes were reported in 20% of patients (14). Different etiologies from different studies are compared and shown in Table 3.

Another common etiology observed in our population was vascular disease by 20%. Vasculopathy was seen in 23.5% of their population study (10).

Coagulopathy disorders as frequent causes were responsible for 20% of strokes in our study. This frequency was higher in comparison with another study from Iran; prothrombotic disorders were showed in 7.5% of cases with stroke (14). However, there is a higher incidence regarding prothrombotic abnormality (50%) (11). 

Although congenital heart disease is reported the main causes of childhood stroke (responsible for 15%-30%), this rate in our study was not notable (6.66%). 

**In conclusion, **the incidence of stroke in children was 0.75 per 100000. Hemorrhagic stroke due to major trauma, coagulopathy and vasculopathy were the most frequent causes that necessitate implementing strategies for prevention, earlier diagnosis, and treatment.
